# Migrants’ human rights and health protection during the COVID-19 pandemic in the Mediterranean Sea: what we have learnt from direct inspections in two Italian hotspots

**DOI:** 10.3389/fpubh.2023.1129267

**Published:** 2023-04-21

**Authors:** Cristoforo Pomara, Salvatore Angelo Zappalà, Monica Salerno, Francesco Sessa, Massimiliano Esposito, Giuseppe Cocimano, Salvatore Ippolito, Alessandro Miani, Eduardo Missoni, Prisco Piscitelli

**Affiliations:** ^1^Legal Medicine, Department of Medical, Surgical and Advanced Technologies, “G.F. Ingrassia”, University of Catania, Catania, Italy; ^2^Member of the Task Force of the Sicily Region for Immigration, Catania, Italy; ^3^Department of Law, University of Catania, Catania, Italy; ^4^Former Officer at the United Nations High Commissioner for Refugees, Geneve, Switzerland; ^5^Italian Society of Environmental Medicine, Milan, Italy; ^6^Department of Health Science and Policy, University of Milan, Milan, Italy; ^7^Global Health Researcher, Bocconi University, Milan, Italy

**Keywords:** COVID-19, migrants, immigration, SARS-CoV-2, hotspot

## Abstract

This study aims to assess the situation of Italian hotspots for migrant reception during the COVID-19 pandemic, and specifically analyzing the situation of two hotspots located in the Sicily Region (Pozzallo harbor and Lampedusa Island), to identify critical issues. At the same time, we hypothesize solutions to guarantee the respect of human rights and suggest an operational protocol to be applied in similar situations, considering that the migration phenomenon is increasing and involving new geographical areas. Based on data obtained through the site inspections, the facilities of Pozzallo and Lampedusa exceeded their capacity to adequately contain the spread of the SARS-CoV-2 infection. Considering these findings, we suggest a practical workflow summarizing the main actions that should be applied to contain COVID-19, or other infectious disease, spreading in hotspots for migrants. The impact of the COVID-19 pandemic on migrants has received limited attention, although the migration phenomenon did not slow down during the pandemic period. Regarding the risk of spreading infectious diseases such as COVID-19, it is necessary that those countries who are most exposed to migration flows, such as Italy, plan dedicated strategies to minimize the possibility of transmission of SARS-CoV-2, using adequate protocols to monitor the possible insurgence of variants of interest (VOIs) or variants of concern (VOCs). Finally, it is important to state that these suggestions could be applied in any future pandemics.

## 1. Introduction

The rule of law and the protection of human rights are essential foundations for a democratic society and social peace. Based on international studies, it has been reported that violations of the human rights of migrants are so widespread and common that they are a distinctive feature of this phenomenon (1). The motivation behind migration has been investigated for many decades, through different approaches, applying different analytical techniques, raising awareness of what drives international migration flows between countries (the macro-level) and of migration decision-making by individuals (the micro-level) as well as of the complexity of the phenomenon (2, 3). Understanding patterns and drivers of migration represents a central concern in politics and policymaking. Moreover, the recent COVID-19 pandemic scenario has raised questions about the health policies that frequently are not adequate for this phenomenon (4, 5). Despite the fact that it is not defined under international law, the term “migrant” is an umbrella term, reflecting the common idea of a person who moves away from his or her Nation across an international border, temporarily or permanently, and for a variety of reasons. The term includes a number of well-defined legal categories of people, such as people who leave home to get an education, migrant workers, or subjects forced to flee persecution, or human rights violations such as torture (6).

The human migration phenomenon has a long history and is likely to continue in the future; particularly, the growth of the world population with related problems represents the most important cause of this event. Changes in the scale and direction of migration flows have led to the emergence of new destination hubs such as Europe, the Arab Gulf, and parts of Asia (2, 7). In many countries, the legal enforcement of human rights is inadequate or lacking, especially for irregular migrants (subjects who arrived from another Nation without permission) (8, 9). Despite the pandemic context, in the first half of 2021, one million people were forced to leave their countries due to conflicts, violence, or gross human rights violations (10). Migrants face several daily challenges to reach hospitable countries, including human rights violations (sexual violence, imprisonment without respect for human rights, and inhuman travel conditions). Outbreaks of disease, in general and specifically the COVID-19 pandemic, pose additional challenges. Nevertheless, as highlighted in the latest 2021 Mid-Year Trends Report, the United Nations High Commissioner for Refugees (UNHCR) estimated that global forced displacement had almost certainly exceeded 84 million by mid-2021, showing a sharp increase from the 82.4 million reported at the end of 2020 (10). Focusing on the Italian scenario, most of the migrants are of African origin, mostly transported illegally. There are reports, in fact, that indicate that 50% of missing/dead migrants recorded in the Mediterranean Sea are of African origin (11).

From the beginning of the COVID-19 pandemic, international attention has been focused on protecting vulnerable subjects, including migrants and refugees, with the aim of safeguarding individual and public health (12). The spread of the COVID-19 infection in the reception centers for migrants and refugees in Europe has been of special concern (13). Inter-governmental co-operation in the “management” of migration is expanding rapidly, with regional inter-governmental consultative processes operating in all European countries, usually focusing on strengthening inter-state cooperation in the control and prevention of irregular migration through improved border checks, information sharing, return agreements, and other measures (14). Considering the pandemic period, the international collaboration to mitigate the responsibilities of countries which – being entry points to the European space – are most exposed to the inflow of migrants was of great importance.

Efforts to promote the respect of migrants’ human rights and contain xenophobia remain fragmented, limited in their real impact, and lacking in allocation of adequate resources (15). According to their capacities and political orientation, governments of the involved countries provide different levels of guidance, services, and assistance to migrants, public education, and advocacy for the respect of migrants’ rights and dignity. Nevertheless, considering the sometimes-limited resources made available and the transnational dimension of the phenomenon, the need emerges for wider initiatives for migrants’ rights and inter-agency cooperation among international organizations (1, 16). The migration phenomenon has been inevitably affected by the situation determined by the still ongoing, though reduced, COVID-19 pandemic (17). The impact of the COVID-19 pandemic on millions of migrants received relatively limited attention, although the migration phenomenon did slow down during the pandemic (10). Indeed, the COVID-19 pandemic and related containment policies have worsened the most relevant pre-pandemic issues: exclusion of migrants from the labor market increased due to new economic difficulties, enforcement of isolation and rejection. In addition, it is important to underline the difficulties in organizing the arrival of migrants, considering that each country has adopted various measures to contain the pandemic risk (screening on arrival, isolation, etc.). These problems are amplified in the case of unaccompanied minors: the number arriving in the European Union (EU) has been increasing dramatically over recent years (18, 19).

The hotspot approach has been one of the key EU responses to the 2015 migration crisis (20). In the pandemic period, hotspots were areas with a high risk of COVID-19 transmission. In fact, considering that frequently the number of migrants was higher compared to the maximum, it did not allow social distancing thus, possibly, increasing the number of infections (21). Indeed, the higher the population density within an environment, such as within a hotspot, the greater the probability of SARS-CoV-2 infection (22–24). One of the substantial problems of the correlation between COVID-19 infection and population density is also based on the consequent ineffectiveness of social isolation as a preventive measure. Since social isolation and safety distance are effective methods of prevention, the increase in population density inevitably results in an obstacle (25). This concept has been confirmed by numerous studies that reported that the high rate of COVID-19 infectivity and transmissibility is related to highly populated and humid environments (26). Furthermore, an increase in the incidence and mortality of COVID-19 has been demonstrated in places where there is a higher number of foreign travelers with poor economic conditions, such as migrants, causing an increase in mortality not only for the foreign community but also for the general population (27–29). Similar results were published in a study conducted in Algeria that showed that the incidence of COVID-19 was greater in areas with higher population densities (30). A study conducted in Thailand confirmed these results by establishing that the infection correlation coefficient was associated with an increase in infection rate (31). Velasco et al., confirmed the same data through a study involving 141 countries affected by COVID-19 (32). This concept was also addressed by Mathur et al. where, through an observational cohort study conducted in the United Kingdom, it was concluded that even in the battle against COVID-19 it is necessary to break down ethnic inequalities, through the elimination of obstacles, equitable healthcare, and an improvement of the diffusion of tests and vaccinations (33).

This paper focused on the Italian situation related to the migration phenomenon, considering that it has been at the center of public debates (34). The Italian system for the reception and integration of asylum seekers, refugees and migrants has been a hot topic over the last few years, even before the pandemic, and legal frameworks have been frequently changed (35, 36). Most recently, in October 2020, with the institution of the SAI (“system of reception and integration”), managed by municipalities and NGOs, which has two levels: the first is dedicated to the international protection of migrants, and the second is devoted to those who have already been granted international protection and need additional integration services. In addition, there is a parallel system for the reception and integration of asylum seekers, the Extraordinary Reception Centers (CAS), coordinated by prefectures (the local representatives of the Government in each Italian province) (37). The hotspots, consisting in hosting facilities usually located in the proximity of landing points, are specific areas controlled by State authorities where all the new arrivals undergo medical screening and receive information concerning immigration and asylum legislation.

Migrants and asylum seekers have been crossing the Mediterranean for decades, generating serious problems in different places where migrants often land, such as Lampedusa, an Italian island. During the pandemic period, the correlation between COVID-19 and migrants generated a serious public health problem that has raised awareness in the entire European Community (38–42). A recent literature review analyzed 77 articles from nine European countries in order to provide a “state of the art” of migrants’ access to health care, underlining that there is a serious disparity in access to hospital environments by this population. The authors conclude that it is necessary to improve health care services for migrants, also breaking down language barriers through specific tools (43).

This study aims to assess the situation of Italian hotspots for migrant reception during the COVID-19 pandemic, and specifically investigating two hotspots located in the Sicily Region (Pozzallo harbor and Lampedusa Island), to identify critical issues. At the same time, we hypothesized different solutions to guarantee the respect of human rights and suggest an operational protocol to be applied in similar situations, considering that the migration phenomenon is constantly increasing and involving new geographical areas.

## 2. Methods

### 2.1. Study area

**Figure 1:**
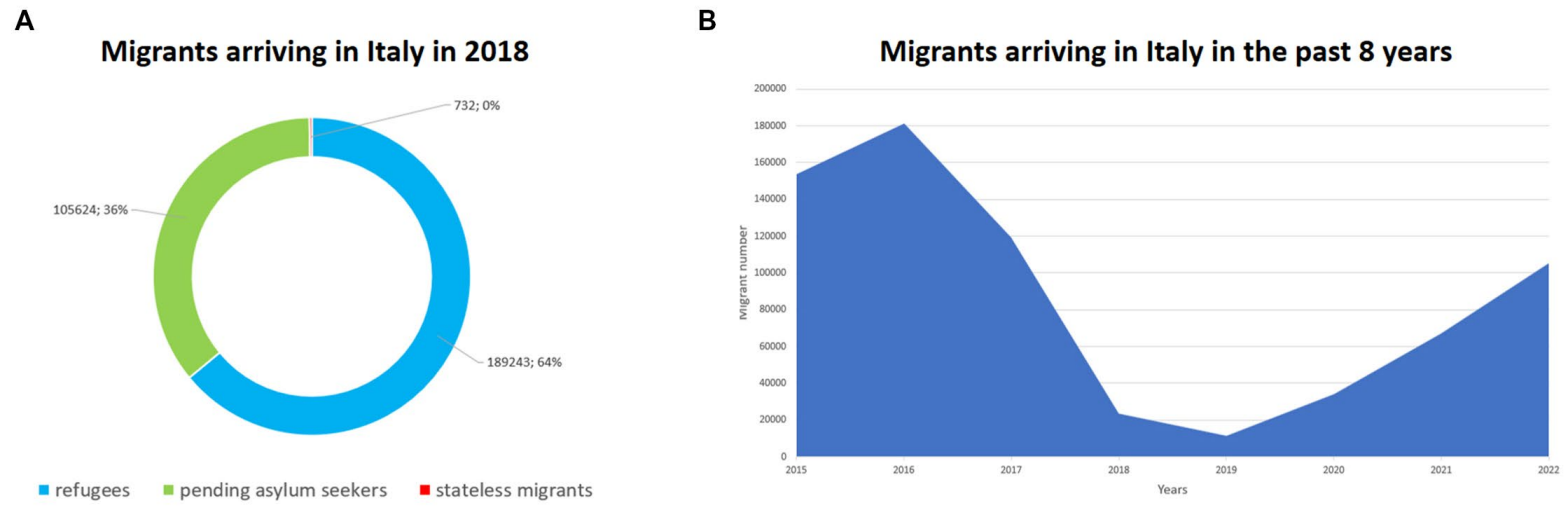
**(A)** Migrant categories arriving in Italy in 2018. **(B)** The trend of migrants arriving in Italy in the last 8  years.

During the recent surge of migrants crossing the Mediterranean Sea in search of protection, Sicily became the second main area of arrival in Europe after Greece. Indeed, based on the UNHCR report, limiting the analysis to the African route, at the end of 2018, a total of 295,599 migrants arrived in Italy (189,243 refugees, 105,624 pending asylum seekers, and 732 stateless migrants) mainly passing through Sicily, 137,757 from Greece, 101,597 from Spain, 10,461 from Malta, 27,321 from Cyprus (Figure 1A).

As summarized in Figure 1B, based on data provided by the Italian Ministry of Internal Affairs concerning the last 8 years, Italy received (as country of first arrival) a total of 153,843 migrants in 2015, 181,436 in 2016; 119,369 in 2017; 23,370 in 2018; 11,471 in 2019; 34,154 in 2020; 67,040 in 2021; and 105,129 in 2022 (the reduction during 2018 and 2019 is related to the different policies of immigration adopted by the Italian Government in the management of immigration).

**Figure 2:**
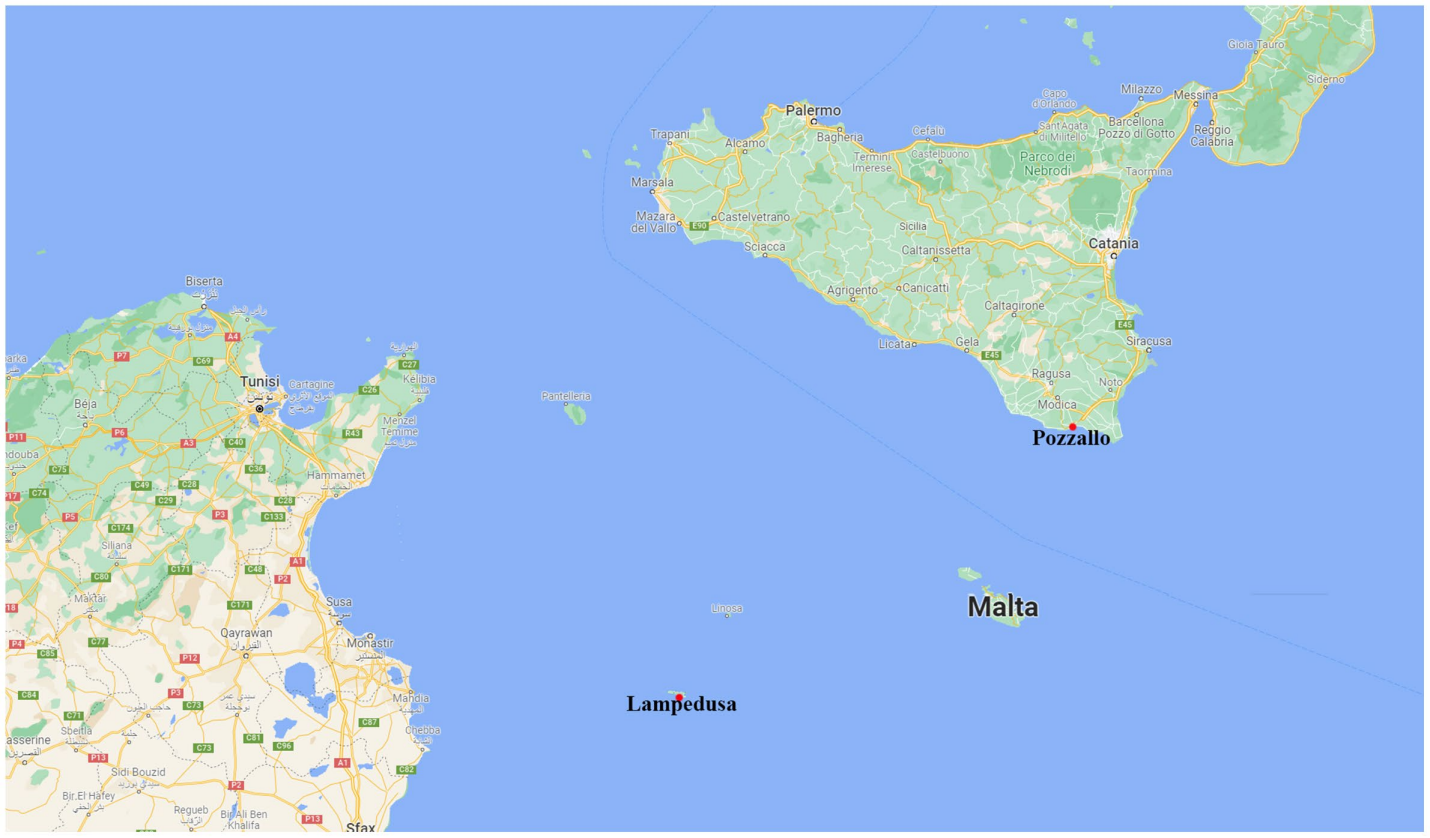
Area affected by the migration route between North Africa and Sicily, in this paper we focused on the situation of two harbors (marked by a red point): Pozzallo and Lampedusa.

Sicily is the largest region of Italy and the largest island in the Mediterranean Sea, but in terms of economic development, it lags far behind the average national and European economic conditions, recording the second-lowest Gross Domestic Product (GDP) *per capita* and Purchasing Power Standards (PPS) among Italian Regions (44). The route between North Africa and Sicily has a long history, starting in the 1990s to supply seasonal workers to Sicilian agriculture. However, the so-called Mediterranean route has become the prevalent migration route towards Italy only since the mid-2000s. There are 8 harbors located in Sicily that are affected by this phenomenon: Pozzallo, Lampedusa, Catania, Augusta, Messina, Trapani, Palermo, and Porto Empedocle. In this paper, we focused on the ports of Pozzallo and Lampedusa (Figure 2).

### 2.2. Italian National framework dealing with asylum seekers and refugees, with a focus on the Region of Sicily

The Italian legislative framework provides that Regional Governments may regulate the social protection of migrants who arrive in their territory. Therefore, hosting policies, as well as access to social and health services, education, and training are carefully planned and carried out in cooperation among national, regional, and local authorities or independently at sub-national government levels. Nevertheless, mainstreaming and integration policies/programs are now implemented throughout the country with no differences between regions (45).

After disembarkation, all foreign citizens who arrive in Italy without permission are assisted; they are identified and retained for expulsion or, in cases of international protection seekers, for the assessment procedure. This usually occurs in the so-called “hotspots,” a system of centers for the rapid identification and classification of migrants’ and refugees’ status that was introduced in 2015 by the European Migration Agenda. Sicilian hotspots are in Pozzallo, Porto Empedocle, Lampedusa, Trapani, Augusta, and Taranto. In each of these hotspots, there are first-reception facilities that can receive around 1,500 people for identification, registration, and fingerprinting purposes.

In this paper, we focus on the site inspections with our participation on 1 September 2020 for the Lampedusa Hotspot, and on 25 August 2020 for the Pozzallo Hotspot, which can receive 234 and 187 migrants, respectively. Indeed, in the period between 01/01/2020 to 25/08/2020, an additional 17,504 migrants arrived in Italy (average number of migrants/day was 73), while from 25/08/2020 to 31/08/2020, a total of 1,690 migrants landed, with an average number of migrants/day equal to about 241.

### 2.3. Hotspot site inspections

To perform the site inspection, we applied the principle of the medical-legal investigation. All team members had to wear the recommended personal protective equipment (PPE). The exact arrival time was recorded, reporting all circumstances (such as weather conditions, indoor/outdoor environmental variables, etc.). We provide a detailed description of the site thanks to photographic documentation and notes. Moreover, we applied laser scanner 3D technologies (Leica BLK 360). Considering the pandemic period, we carefully evaluated the respect of the regulations to contain the risk of COVID-19 infection. In this regard, the European Centre for Disease Prevention and Control (ECDC) published specific guidelines aimed at providing scientific advice on public health principles and considerations for infection and prevention control of COVID-19 in migrant and refugee reception and detention centers (46). Based on the ECDC documents, we evaluated the management of the following points:

- the application of quarantine in reception and detention centers;
- the preparedness of the settings of migrant reception and detention, particularly in cases of a sudden influx of migrants, addressing seven key dimensions: human resources, medicines and vaccines, physical infrastructure, sanitation and hygiene, health financing, coordination, and health information;
- screening for infectious and chronic diseases, focusing on COVID-19;
- living conditions in settings of migrant reception and detention;
- management of possible, probable, or confirmed COVID-19 cases in reception and detention settings.

This study is based on operations requested by the Regional Government of Sicily to the Task Force for immigration of the Sicily Region, to provide recommendations aimed at containing the risk of the spread of the SARS-CoV-2 infection. All data collected were treated anonymously. This was the first step in providing information concerning the risks of pandemic spread in migrant centers. The approval of the Institutional Review Board and Ethical Committee was not required since it is not necessary based on the current Italian legislation; moreover, all activities were performed under National emergency status. All procedures were performed in accordance with the declaration of Helsinki.

## 3. Results

### 3.1. Lampedusa hotspot

**Figure 3:**
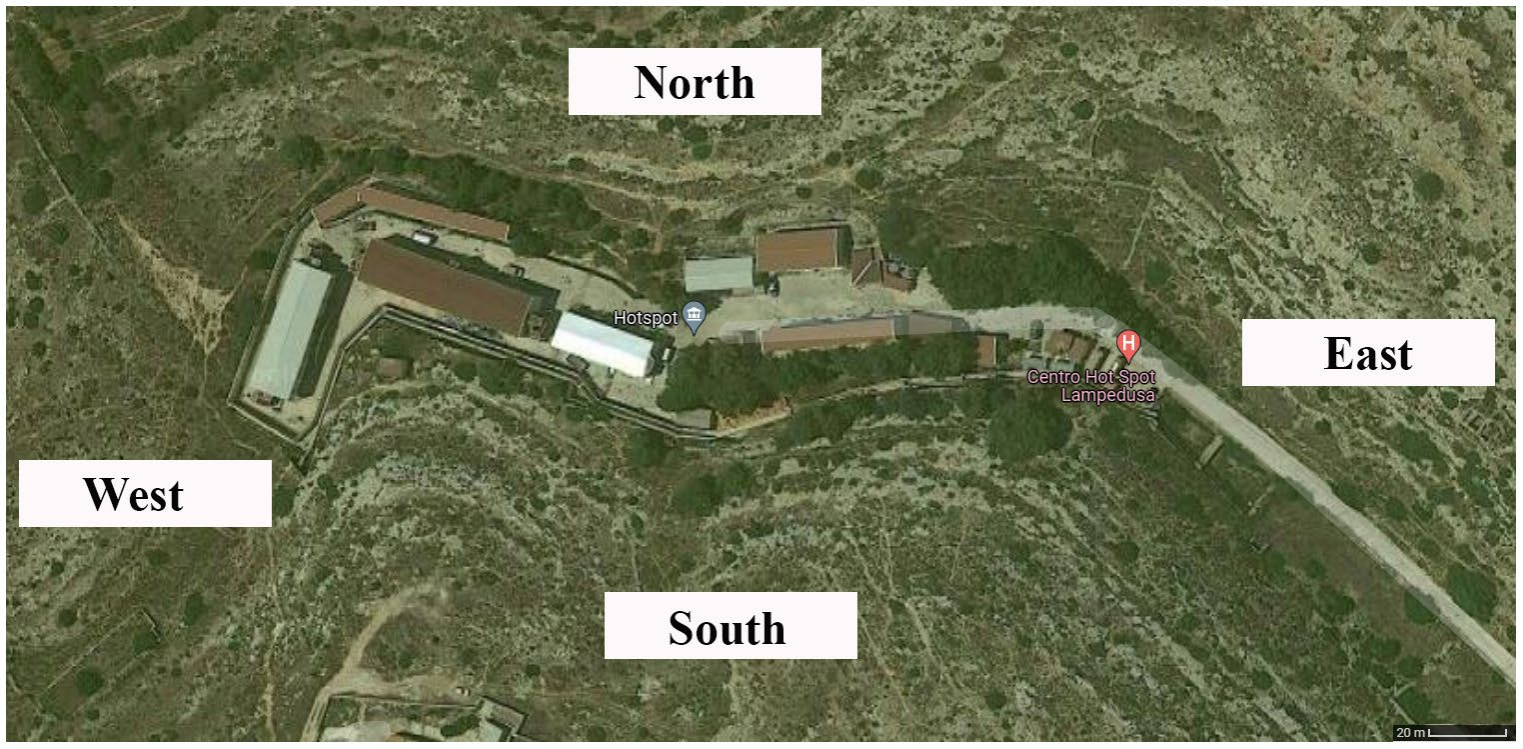
Satellite image of Lampedusa hotspot; source: geographical internet service (available at: https://www.google.it/maps/@35.5112635,12.6007617,229m/data=!3m1!1e3).

On 1 September 2020, at 09:30, an inspection was carried out at the hotspot located on Lampedusa Island. In Figure 3, the hotspot area is shown.

Just beyond the gate, immediately to the left of the entrance, there was a building used by the Italian Army. At the entrance, a sanitizing liquid dispenser was present. Personnel belonging to different military corps (Italian Army, Guardia di Finanza, State Police) and the Italian Red Cross were present, with patrol cars and ambulances. At the right side of the entrance, there were 2 terraces on which groups of migrants were found. These migrants were partly seated and partly lying on sponge mattresses; they were separated from each other by emergency tape according to the date of disembarkation and arrival at the hotspot, hand-written on a sheet of A4 paper hanging on the emergency tape. Only a few migrants wore surgical masks.

Crossing the main road, we found other migrants grouped together and not respecting social distancing, and again only a few migrants wore surgical mask. The central building was in poor hygienic conditions; also, the medical room was untidy and without adequate space.

To reach the upper floor, it was necessary to use external iron stairs on the west side of the building. We found seven messy dormitory rooms in poor hygienic conditions, with bunk-beds arranged in rows. The space between beds and rows was not adequate, not having the prescribed distancing. Two toilets in very poor physical and hygienic conditions were found; the use of the toilet was very problematic, due to the presence of rubbish scattered on the floor.

Subsequently, we explored the rear of the main building, observing numerous migrants in the external area, on mattresses, blocking free movement. Moreover, we visited 2 prefabricated buildings used as storage facilities, containing foodstuffs in poor condition, arranged disorderly. Continuing our visit, we arrived at a two-storey building, bordered by grey metal railings, and delimited by emergency tape, used to isolate SARS-CoV-2 positive migrants.

Continuing along the main road, we visited the kitchen building, which we found in good order and adequate hygienic conditions. Moreover, we visited another two-storey building, but only the ground floor was accessible. This building had rooms used as dormitories and toilets in very poor condition. Finally, it is important to remark the presence of numerous migrants in the external areas without adopting any prevention measure to contain the spread of the SARS-CoV-2 infection (no facemasks, no social distancing); some of them were lying on sponge bed mattresses.

### 3.2. Medical-legal considerations about the Lampedusa hotspot

In light of this site inspection, different actions should be taken to improve the healthcare conditions for migrants. As previously discussed, the hotspot of Lampedusa currently accommodates an epidemiologically “mixed” population, hosting, at the same time, individuals who tested positive and negative for SARS CoV-2. Moreover, other infective conditions both spread by contact (e.g., scabies) and the respiratory route (e.g., tuberculosis) were not evaluated, despite overpopulation representing a risk factor. It is important to say that the hotspot was inspected in a moment of emergency, considering the number of landings in that period. To date, due to the current structural settings of the housing modules, the optimum reception capacity is set at about 187 migrants; on the other hand, at the time of inspection, the number of migrants was 5 times higher than the above-mentioned suitable number of migrants that the hotspot should accommodate.

Moreover, considering the geographical conformation of the hotspot area, only partial risk mitigation measures may be applied, for example, hosting numerous migrants in an open area, despite the fact that this situation improves the risk of crowding. Finally, the daily number of SARS-CoV-2 tests (about 200/day) was not adequate for monitoring the health status of migrants, considering that the situation changes quickly, day by day, with new arrivals and new departures. Taking into account three factors, (a) time to perform the molecular tests, (b) the need to house migrants in promiscuous areas while waiting for test results, and (c) the hosting time, which can extend to more than 15 days, testing becomes useless. In these conditions, if a positive subject is not rapidly diagnosed, he/she may spread infection. Obviously, the same risk is represented by false negatives due to the sensitivity of the method.

### 3.3. Pozzallo hotspot

**Figure 4:**
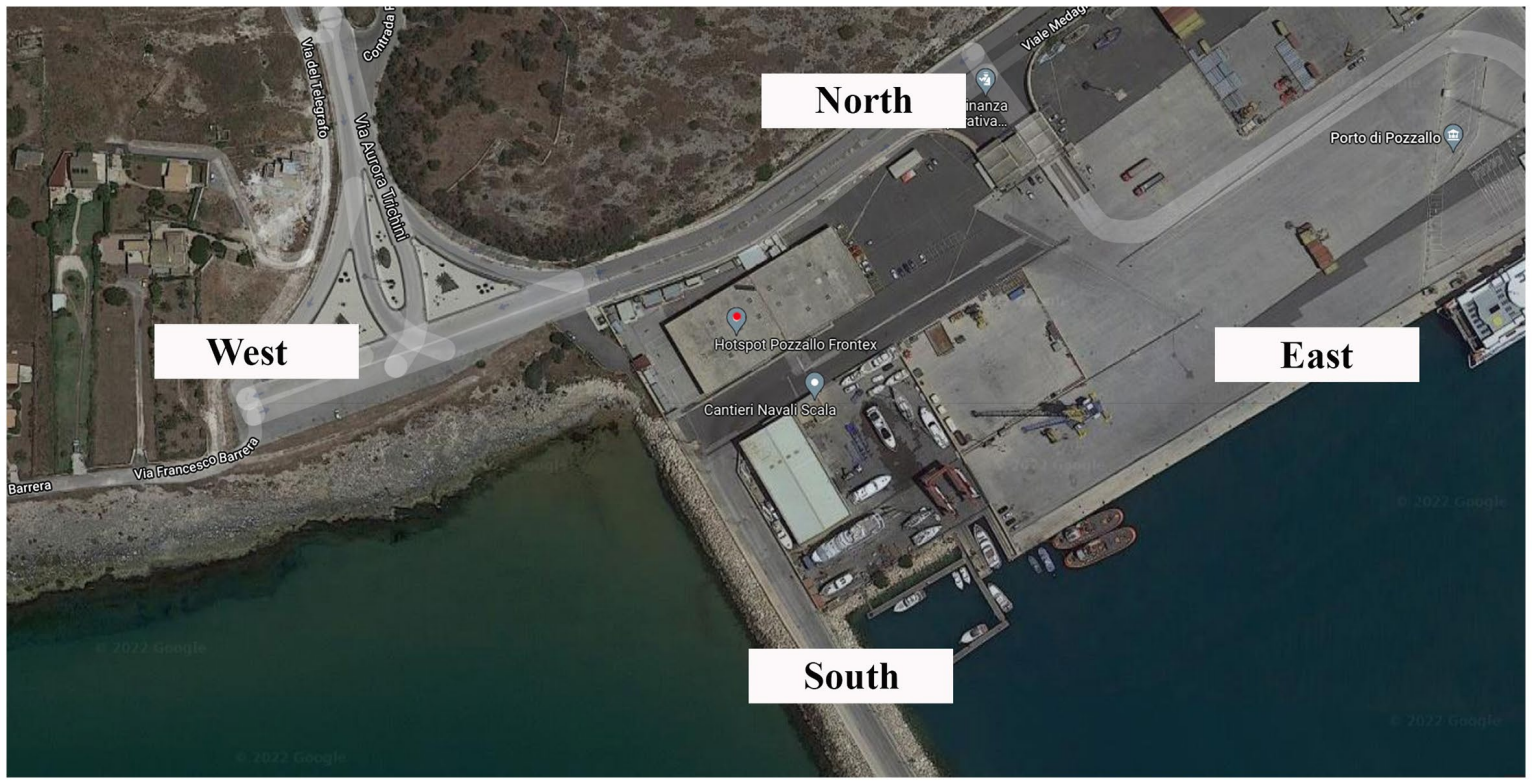
Satellite image from the geographical internet service (available at: https://www.google.com/maps/@36.7152598,14.8265993,361m/data=!3m1!1e3).

On 25 August 2020, an inspection was carried out at the hotspot located in Pozzallo (Southern Sicily). In Figure 4 the hotspot area is shown.

The Hotspot area was separated from the external area by a closed metal gate. The hotspot was composed of a low building, with a prominent paved area. Beyond the gate there were 6 prefabricated buildings used as offices, in the external area, there was an ambulance guarded by soldiers. Placed in front of the prefabricated buildings, along the southwest side of the main building, there were 3 field tents used by the Civil Defense, delimited by metal barriers and emergency tape. The main road between the northwest side of the main building and the offices led to a small closed area.

**Figure 5:**
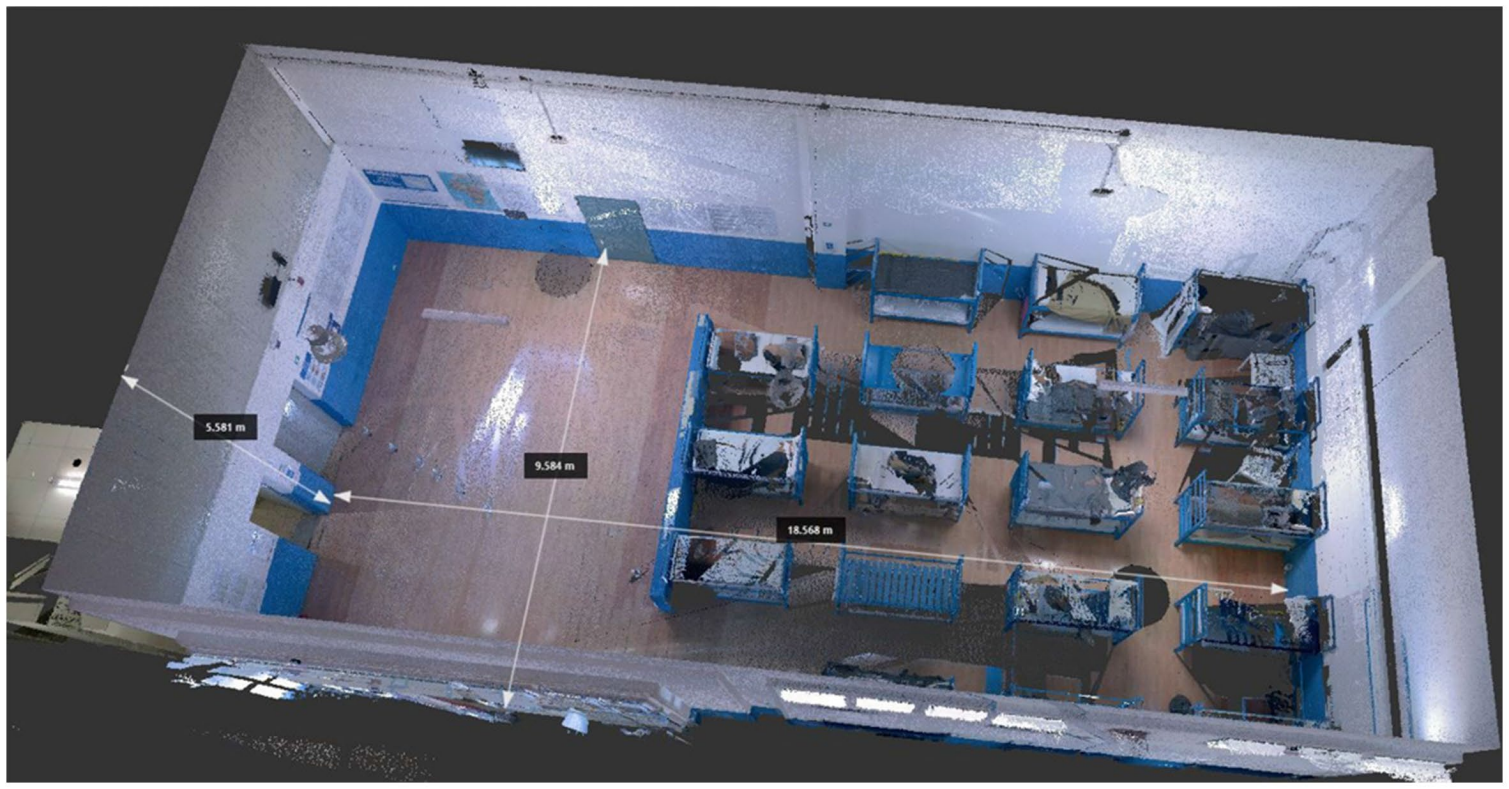
3D reconstruction of hotspot in Pozzallo obtained with a Leica BLK 360. The room measured 18.568  ×  9.584  ×  5.581 m.

It was possible to access the dormitory through a door (Figure 5), which was untidy and in poor hygienic conditions, where 3 rows of 12 bunk-beds were found. At the entrance of the dormitory, two doors led to the toilets, which were subdivided into two zones. The first zone was composed of 1 toilet for disabled people (toilet, shower, washbasin) and 2 washbasins, 3 showers, and 3 toilets. The other room had 6 toilets, 3 washbasins, and 6 showers.

Going outside, along the same side as the dormitory entrance, there was another door with access to a room used as an office: there was a desk, a plastic chair, a bench, 2 pieces of metal furniture, and 1 wooden table; on the other side, there were 4 plastic chairs and 1 metal cabinet. Through a door it was possible to access a room similar to an office, with a desk and chairs. Through this room, we arrived in a large room used as a dormitory. Inside the dormitory, there were numerous migrants, without social distancing and facemasks. In the same room, there was a toilet for migrants. Nearby, there was a staff toilet, divided for males and females. Proceeding along the same side of the building, there was another door, which was open and gave access to a service room. Moreover, there was a metallic door, which was ajar and blocked only by a van of the Carabinieri.

In the posterior area of the main building, there were makeshift tents made by migrants, which appeared delimited and closed by a blue metal structure. Walking along the southeast side of the building, there was a door, flanked by a gate identical to the ne on the northwest side already described, which appeared to be blocked by a wood and metal platform.

Going on, there was another double door, which was open, with a sign saying, “entrance forbidden … danger of intoxication.” Through this door it was possible to access the infirmary, which was in excellent condition as regards order and hygiene. Moreover, there was a delimited area with a door; on the door there were two notices: the first one recommended the use of PPE, the second one recommended paying attention to the intoxication risk. Through this door, it was possible to arrive in a second dormitory, in good condition as regards order and hygiene, with 3 rows of 4 beds and 2 rows of 3 metal bunk-beds. At the rear of this dormitory, there were two metal doors, which gave access to the toilets, divided into two separate zones: the first area was equipped with 3 toilets, 3 washbasins, and a separate room with 1 washbasin and 1 toilet. The other area was divided into two different rooms, one equipped with 3 showers, 1 toilet, 1 washbasin, the other one with 3 toilets and 1 washbasin.

### 3.4. Medical-legal considerations about the Pozzallo hotspot

Based on the data obtained through the site inspection, the facilities of the Pozzallo hotspot did not offer receptivity adequate to contain the spread of the SARS-CoV-2 infection, considering the need to manage an epidemiologically “mixed” population. As previously described, it is important to remark that these critical points may be extended to other diffusive clinical conditions. In this regard, it was not clear if specific clinical management regarding non-SARS-CoV2 pathologies were adopted (scabicidal treatment, chest X-ray). Moreover, the Pozzallo hotspot appeared structurally unsuitable for hosting individuals with the SARS-CoV-2 infection and/or other transmissible infections (transmitted by contact or the respiratory route), considering the high degree of promiscuity, due to the structural settings of the housing modules. The number of migrants was excessive for the internal and external areas, with the impossibility to respect the basic measures to contain the spread of the SARS-CoV-2 infection. In this way, a further concern is related to health surveillance both for migrants and for healthcare personnel: it should be noted that medical personnel were not subjected to a medical examination, nor to surveillance (execution of swabs, serological tests, etc.) for SARS-CoV-2 prevention. Moreover, the healthcare facilities were not adequate to maintain essential hygienic conditions: there were no soap dispensers, no dispensers for sanitizing liquids, and no partition walls in the living/dormitory areas. Another important critical point is related to the absence of the “grey zone” to allow for the isolation of close contacts of SARS-CoV-2 positive subjects. Furthermore, toilets were not adequate for the number of users, without distinction among suppliers, transporters, and other external personnel (i.e., military personnel). There was an absence of dressing/undressing rooms properly equipped with sanitizing solutions and waste containers.

The daily cleaning and periodic sanitizing of rooms, common areas, and recreational areas was not adequate for the number of migrants. Furthermore, the emergency facilities were not sufficient for the number of migrants and staff. Moreover, the hotspot personnel were exposed to a high risk of SARS-CoV-2 infection, considering the number of migrants and the environmental conditions (i.e insufficient ventilation).

In these conditions, the presence of a large group of migrants in the same space for a prolonged period increased the psychological risks: it is important to remark on the absence of adequate linguistic and psychological support to minimize these risks and favor the integration process. Moreover, psychological support should be programmed for all personnel involved in migrant hospitality considering that these operators are exposed to a high risk of burnout.

## 4. Discussion

Migrant management is a critical issue both at the national and international level. The impact of the COVID-19 pandemic on the millions who have been forced to migrate for safety or economic purposes has received too little attention. This situation has been worse for people who live in immigration facilities or refugee camps. In this scenario, Italy is one of the European countries most impacted by this phenomenon, which is facilitated by some factors including: the pressure to migrate, the agility of the network and the total cost of migration (47). It has been demonstrated that a supra-national organization approach at the regional level is the most viable approach in the long term, with the ultimate goal of inter-regional cooperation on migration management based on equality between countries (48).

The COVID-19 pandemic was an event that greatly altered living habits across the board. Subsequent measures put in place to contain the epidemic added to the negative impact on migration policies (49). However, some countries implemented inclusive policies, such as Portugal’s decision to grant full citizenship rights to all migrants and refugees for the duration of the pandemic to ensure that they could freely access health care, testing, and vaccinations. The Spanish government also implemented openness policies, releasing detainees from migrant detention centers to mitigate the risk of the COVID-19 infection (17). These kinds of decisions remained an exception, as most European countries used the pandemic to provide reasons for increased border controls and exclusionary measures targeting refugees. From both a public health and human rights point of view, there are strong reasons to argue that the migration policies adopted in Europe have been both unsuccessful in containing the infection and detrimental to the human rights and welfare of migrants and refugees (50).

During 2016–17, specific national guidelines were published in Italy to provide evidence-based recommendations on healthcare for migrants and asylum seekers upon their arrival in Italy. Based on this document, signs, and symptoms of specific diseases should have been actively searched for such as: active tuberculosis (TB), malaria, sexually transmitted infections (STI), intestinal parasites, diabetes, and anemia. Moreover, for each migrant, different health tests should be performed even in asymptomatic subjects, in order to discover latent forms of TB, HIV, HBV, HCV, STI, strongyloides, schistosoma, and diabetes, particularly when he/she comes from endemic areas, or he/she has been exposed to risk factors. Migrant women are strongly encouraged to perform a pregnancy test, cervical cancer screening, and adhere to relative vaccination programs (51). Despite the COVID-19 spread, these guidelines were not updated, however, the recommendation to contain the risks of the SARS-CoV-2 infection was included.

Moreover, as we have reported, migrants live in inadequate spaces with limited resources to mitigate both the spread of the SARS-CoV-2 infection and other diseases. In agreement with Orcutt et al., asylum seekers in detention centers or refugees in camps were exposed to a higher risk of the SARS-CoV-2 infection due to cramped and unsanitary living quarters. In the same paper, published in May 2020, the authors suggested that several countermeasures should be adopted to contain the spread of the COVID-19 infection, such as: universal and equitable access to health systems; inclusion of migrants and refugees in health protection programs; responsible, transparent, and migrant-inclusive public information strategies (52).

The Italian Government only produced guidelines for the access and stay of migrants in migrant centers in July 2020 (53). In accordance with this document, the migrant will have to undergo a nasopharyngeal swab and, if positive for SARS-CoV-2, will have to be placed in isolation in a dedicated and separate accommodation until there is a double negative swab result, then the migrant will be able to return to the community. Furthermore, if the migrant is a close contact with a positive COVID-19 subject, the migrant must be placed in preventive quarantine and subsequently subjected to a nasopharyngeal swab. Thanks to the help of vaccines and a better knowledge of the virus, unfortunately, these guidelines published in July 2020 are inadequate. The present study serves precisely to contribute to an improvement and update of our knowledge on the subject of COVID-19 and migrants.

Despite the importance of migrant management to contain the spread of the SARS-CoV-2 infection, there is a shortage of knowledge about self-protection measures for contagious diseases and in the perception of the pandemic risk among migrants. Considering the low resources in which the reception centers for migrants and refugees operate, it is important to highlight the importance of the adoption of basic measures to contain the spread of infection, such as implementing social distancing strategies, ensuring the systematic use of PPE, and active syndromic surveillance (54). In our experience, many critical areas are present in the Italian reception system for asylum seekers and refugees. To improve the migrants’ conditions, we suggest to:

- adopt “dockside diagnosis,” performing a screening test for the SARS-CoV-2 infection with a rapid antigenic test for each migrant, confirming all positive/doubtful tests with a molecular test;
- create a “grey” zone to isolate close contacts of positive subjects in order to contain the spread of infection;
- provide extra and dedicated personal and collective hygiene measures for positive subjects (including povidone iodine mouthwash) (55);
- test each negative subject for a period of 5 days;
- avoid the overcrowding of hotspots, favoring the rapid distribution (permanence no longer than 72 h) in other dedicate structures, guaranteeing the respect of indications against the spread of the SARS-CoV-2 infection;
- improve the sanitary facilities both for migrants and for personnel involved in assistance;
- improve the internal environmental conditions, favoring air ventilation and avoiding crowding, particularly in dormitories;
- adopt SARS-CoV-2 immunization, according to the country’s vaccination schedule;
- improve flooring with special materials in order to facilitate washing and disinfection, particularly for migrant bathrooms;
- program two daily cleanings and periodic sanitization of rooms, dormitories, and shared areas;
- improve healthcare for the personnel involved in migrant management, programming medical screening, and medical surveillance for optimizing employee health;
- follow public health measures (social distancing, proper hand hygiene, and self-isolation) to mitigate the risks of infection.

**Figure 6:**
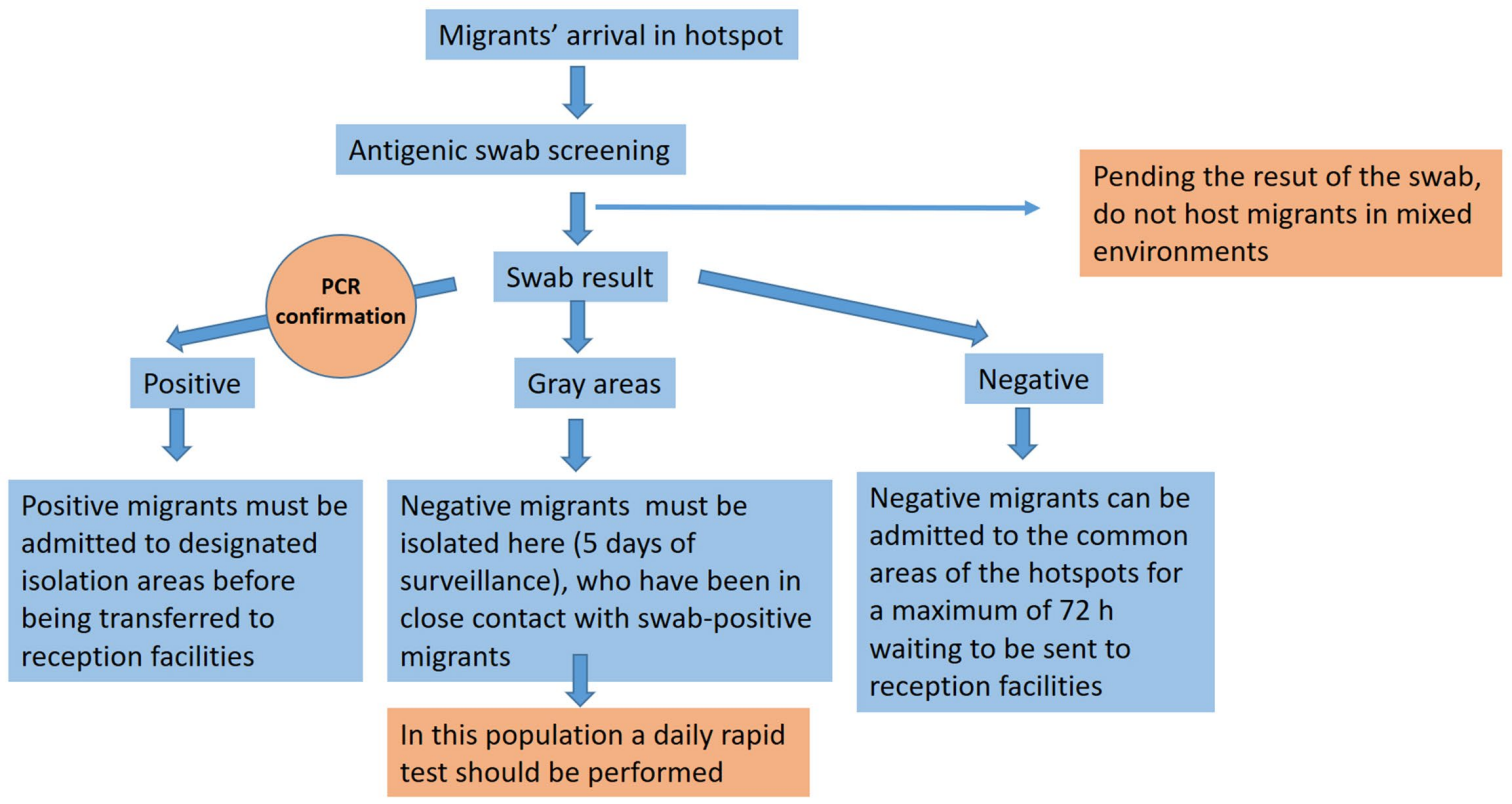
This graph shows a practical workflow that we suggest applying when migrants arrive in order to contain the spread of the COVID-19 infection.

In Figure 6, we summarize the main actions that should be applied to contain the spread of the SARS-CoV-2 infection.

Despite the fact that at the time of site inspection the vaccination against COVID-19 was not available, another crucial point in the containment of the risk of the SARS-CoV-2 infection is the introduction of the vaccination program for migrants. As established in the guidelines for all pathologies that have an approved vaccine, in the absence of valid documentation, immunization should be carried out (51). In the same manner, we encourage the anti-SARS-CoV-2 vaccination for adult migrants. Finally, an important concern related to the current COVID-19 endemic state is the monitoring program of SARS-CoV-2 variants: indeed, the global systems to detect signals of potential variants of interest (VOIs) or variants of concern (VOCs) remain critical (56, 57). In this way, the rapid identification of subjects who tested positive for COVID-19 infection should be combined with molecular investigations in order to identify VOIs and VOCs promptly.

## 5. Conclusion

In cases of epidemic/pandemic emergencies, it is important to rapidly activate containment procedures based on systematic screening, isolation, quarantine, and adopt appropriate hygienic measures, taking into consideration the limitations of contact tracing within a closed community such as that in hotspots for migrants. However, as reported in this article, the reception system was not able to ensure the proper management of migrants, especially in relation to overcrowding due to the huge number of new landings. Moreover, it is fundamental to guarantee the continuity of care, prevention, and health promotion across all reception phases. While ethical questions arise about the need to ensure “a safe harbor” for migrants, practical issues related to the reception system should be considered, such as the respect of basic human rights, health protection and adequate provision of healthcare. Regarding the risk of spreading infectious diseases such as COVID-19, it is necessary that those countries who are most exposed to migration flows, such as Italy, plan dedicated strategies to minimize the possibility of transmission of SARS-CoV-2 by focusing on preventive measures to be applied both upon arrival and continued with monitoring of migrants, rapid sorting and transfer to other centers in order to avoid overcrowding. Moreover, it is important to monitor the possible insurgence of variants of interest (VOIs) or variants of concern (VOCs). All these suggestions should be applied in any possible future pandemics.

## Data availability statement

The original contributions presented in the study are included in the article/supplementary material, further inquiries can be directed to the corresponding author.

## Author contributions

CP, SZ, MS, and PP: conceptualization. CP, SZ, MS, FS, ME, GC, SI, AM, EM, and PP: data collection, data analysis, resources, validation, interpretation, and project administration. ME, FS, and MS: writing—original draft preparation. CP and PP: writing—review and editing. All authors contributed to the article and approved the submitted version.

## Conflict of interest

The authors declare that the research was conducted in the absence of any commercial or financial relationships that could be construed as a potential conflict of interest.

## Publisher’s note

All claims expressed in this article are solely those of the authors and do not necessarily represent those of their affiliated organizations, or those of the publisher, the editors and the reviewers. Any product that may be evaluated in this article, or claim that may be made by its manufacturer, is not guaranteed or endorsed by the publisher.

## References

[1] Taran PA. Human rights of migrants: challenges of the new decade. Int Migr. (2001) 38:7–51. doi: 10.1111/1468-2435.00141

[2] European Commission’s science and knowledge service. International Migration Drivers. A Quantitative Assessment of the Structural Factors Shaping Migration. Baltimore, MD: JRC (2018).

[3] de Haas H. A theory of migration: the aspirations-capabilities framework. Comp Migr Stud. (2021) 9:8. doi: 10.1186/s40878-020-00210-4, PMID: 33680858 PMC7902564

[4] OECD Policy Responses to Coronavirus (COVID-19). The territorial impact of COVID-19: Managing the crisis across levels of government. (2020). Available at: https://www.oecd.org/coronavirus/policy-responses/the-territorial-impact-of-covid-19-managing-the-crisis-across-levels-of-government-d3e314e1/ (Accessed March 31, 2023)

[5] Sahin-Mencutek Z Barthoma S Gökalp-Aras NE Triandafyllidou A. A crisis mode in migration governance: comparative and analytical insights. Comp Migr Stud. (2022) 10:12. doi: 10.1186/s40878-022-00284-2, PMID: 35371921 PMC8934758

[6] Amnesty International. Refugees, Asylum Seekers and Migrants. Website. (2023). Available at: https://www.amnesty.org/en/what-we-do/refugees-asylum-seekers-and-migrants/ (Accessed March 27, 2023).

[7] Czaika M Reinprecht C. Migration drivers: why do people migrate? In: Scholten P, editor. Introduction to Migration Studies: An Interactive Guide to the Literatures on Migration and Diversity. Cham: Springer International Publishing (2022). 49–82.

[8] Koser K. Irregular migration, state security and human security a paper prepared for the policy analysis and research Programme of the global commission on international migration and does not represent the views of the global commission on international migration. Grand-Saconnex: International Organization for Migration (2005).

[9] Aksoy CG Poutvaara P. Refugees’ and irregular migrants’ self-selection into Europe. J Dev Econ. (2021) 152:102681. doi: 10.1016/j.jdeveco.2021.102681

[10] United Nations High Commissioner for Refugees. MID-YEAR TRENDS 2021 Trends at a glance. Mid-year trends report. (2021). 24. Available at: https://www.unhcr.org/statistics/unhcrstats/618ae4694/mid-year-trends-2021.html (Accessed February 16, 2022).

[11] Sagan C. Psychosocial experiences of African migrants in six European Countries. Mix Method Stud. (2020). 10. doi: 10.1007/978-3-030-48347-0

[12] Barron GC Laryea-Adjei G Vike-Freiberga V Abubakar I Dakkak H Devakumar D. Safeguarding people living in vulnerable conditions in the COVID-19 era through universal health coverage and social protection. Lancet Public Health. (2022) 7:e86–92. doi: 10.1016/S2468-2667(21)00235-8, PMID: 34906331 PMC8665842

[13] Sisti LG Di Napoli A Petrelli A Rossi A Diodati A Menghini M. COVID-19 impact in the Italian reception system for migrants during the Nationwide lockdown: a national observational study. Int J Environ Res Public Health. (2021) 18:12380. doi: 10.3390/ijerph182312380, PMID: 34886106 PMC8656734

[14] European Union. The European Union’s cooperation with Africa on migration. (2015) Available at: https://ec.europa.eu/commission/presscorner/detail/fr/MEMO_15_4832 (Accessed February 16, 2022).

[15] Collyer M De Haas H. Developing dynamic categorisations of transit migration. Popul Space Place. (2012) 18:468–81. doi: 10.1002/psp.635

[16] Swepston L Taran P Cholewinski R. Migration, human rights and governance In: Handbook for Parliamentarians (2015). 1–196.

[17] Libal K Harding S Popescu M Berthold SM Felten G. Human rights of forced migrants during the COVID-19 pandemic: an opportunity for mobilization and solidarity. J Hum Rights Soc Work. (2021) 6:148–60. doi: 10.1007/s41134-021-00162-4, PMID: 33758779 PMC7973803

[18] Orsini G Rota M Uzureau O Behrendt M Adeyinka S Lietaert I. Loops of violence(s) within Europe’s governance of migration in Libya, Italy, Greece, and Belgium. Politics Gov. (2022) 10:256–66. doi: 10.17645/pag.v10i2.5183

[19] Barn R Di Rosa RT Kallinikaki T. Unaccompanied minors in Greece and Italy: an exploration of the challenges for social work within tighter immigration and resource constraints in pandemic times. Soc Sci. (2021) 10:134. doi: 10.3390/socsci10040134

[20] Loschi C Slominski P. The EU hotspot approach in Italy: strengthening agency governance in the wake of the migration crisis? J Eur Integr. (2022) 44:769–86. doi: 10.1080/07036337.2022.2047186

[21] Nakada LYK Urban RC. COVID-19 pandemic: environmental and social factors influencing the spread of SARS-CoV-2 in São Paulo. Environ Sci Pollut Res. (2021) 28:40322–8. doi: 10.1007/s11356-020-10930-w, PMID: 32989697 PMC7521763

[22] Arif M Sengupta S. Nexus between population density and novel coronavirus (COVID-19) pandemic in the south Indian states: a geo-statistical approach. Environ Dev Sustain. (2021) 23:10246–74. doi: 10.1007/s10668-020-01055-8, PMID: 33144832 PMC7596317

[23] Benita F Gasca-Sanchez F. The main factors influencing COVID-19 spread and deaths in Mexico: a comparison between phases I and II. Appl Geogr. (2021) 134:102523. doi: 10.1016/j.apgeog.2021.102523, PMID: 34334843 PMC8313543

[24] Magd H Asmi K Henry K. COVID-19 influencing factors on transmission and incidence rates-validation analysis. J Biomed Environ Sci. (2020) 1:277–91. doi: 10.37871/jbres1155

[25] Wong DWS Li Y. Spreading of COVID-19: density matters. PLoS One. (2020) 15:e0242398. doi: 10.1371/journal.pone.0242398, PMID: 33362283 PMC7757878

[26] Diao Y Kodera S Anzai D Gomez-Tames J Rashed EA Hirata A. Influence of population density, temperature, and absolute humidity on spread and decay durations of COVID-19: a comparative study of scenarios in China, England, Germany, and Japan. One Health. (2021) 12:100203. doi: 10.1016/j.onehlt.2020.100203, PMID: 33344745 PMC7736723

[27] Jabłońska K Aballéa S Toumi M. Factors influencing the COVID-19 daily deaths’ peak across European countries. Public Health. (2021) 194:135–42. doi: 10.1016/j.puhe.2021.02.037 33892351 PMC7980183

[28] Jin J Agarwala N Kundu P Harvey B Zhang Y Wallace E. Individual and community-level risk for COVID-19 mortality in the United States. Nat Med. (2021) 27:264–9. doi: 10.1038/s41591-020-01191-8, PMID: 33311702

[29] Tanne JH Hayasaki E Zastrow M Pulla P Smith P Rada AG. Covid-19: how doctors and healthcare systems are tackling coronavirus worldwide. BMJ. (2020) 368:m109. doi: 10.1136/bmj.m1090, PMID: 32188598

[30] Kadi N Khelfaoui M. Population density, a factor in the spread of COVID-19 in Algeria: statistic study. Bull Natl Res Cent. (2020) 44:138. doi: 10.1186/s42269-020-00393-x, PMID: 32843835 PMC7439635

[31] Tantrakarnapa K Bhopdhornangkul B Nakhaapakorn K. Influencing factors of COVID-19 spreading: a case study of Thailand. J Public Health. (2022) 30:621–7. doi: 10.1007/s10389-020-01329-5, PMID: 32837844 PMC7301627

[32] Velasco JM Tseng W-C Chang C-L. Factors affecting the cases and deaths of covid-19 victims. Int J Environ Res Public Health. (2021) 18:1–10. doi: 10.3390/ijerph18020674, PMID: 33466900 PMC7829817

[33] Mathur R Rentsch CT Morton CE Hulme WJ Schultze A MacKenna B. Ethnic differences in SARS-CoV-2 infection and COVID-19-related hospitalisation, intensive care unit admission, and death in 17 million adults in England: an observational cohort study using the OpenSAFELY platform. Lancet. (2021) 397:1711–24. doi: 10.1016/S0140-6736(21)00634-6, PMID: 33939953 PMC8087292

[34] Mariani RD Pasquini A Rosati FC. The Immigration puzzle in Italy: a survey of evidence and facts. Ital Econ J. (2023) 9:85–116. doi: 10.1007/s40797-021-00168-x

[35] Immordino P Genovese D Morales F Casuccio A Amodio E. Epidemiological characteristics of COVID-19 cases in non-Italian nationals in Sicily: identifying vulnerable groups in the context of the COVID-19 pandemic in Sicily, Italy. Int J Environ Res Public Health. (2022) 19:5767. doi: 10.3390/ijerph19095767, PMID: 35565161 PMC9105146

[36] Petrelli A Di Napoli A. The impact of COVID-19 on the immigrant population in Italy. Context, methodology and synthesis of the main evidence from the project of the National Institute for health, migration and poverty (INMP) and Italian regions | L’impatto del COVID-19 nella pop. Epidemiol Prev. (2022) 46:7–13. doi: 10.19191/EP22.4S1.051, PMID: 35862555

[37] European Commission. Italy: How has the reception system for asylum seekers and refugees changed? (2021). Available at: https://ec.europa.eu/migrant-integration/news/italy-how-has-reception-system-asylum-seekers-and-refugees-changed_en (Accessed February 23, 2022).

[38] Giliberti L Queirolo PL. The hole, the corridor and the landings: reframing Lampedusa through the COVID-19 emergency. Ethn Racial Stud. (2022) 45:1760–81. doi: 10.1080/01419870.2021.1953558

[39] Newland K. Will International Migration Governance Survive the COVID-19 Pandemic? Washington, DC: Migration Police Institute (2020).

[40] Mitchell K Sparke M. Hotspot geopolitics versus geosocial solidarity: contending constructions of safe space for migrants in Europe. Environ Plan D. (2018) 38:1046–66. doi: 10.1177/0263775818793647

[41] Lindert J Schouler-Ocak M Heinz A Priebe S. Mental health, health care utilisation of migrants in Europe. Eur Psychiatry. (2008) 23:s114–20. doi: 10.1016/S0924-9338(08)70057-9 18371575

[42] Tazzioli M. Containment through mobility: migrants’ spatial disobediences and the reshaping of control through the hotspot system. J Ethn Migr Stud. (2018) 44:2764–79. doi: 10.1080/1369183X.2017.1401514

[43] Lebano A Hamed S Bradby H Gil-Salmerón A Durá-Ferrandis E Garcés-Ferrer J. Migrants’ and refugees’ health status and healthcare in Europe: a scoping literature review. BMC Public Health. (2020) 20:1039. doi: 10.1186/s12889-020-08749-8, PMID: 32605605 PMC7329528

[44] ISTAT. ISTAT (2022). Available at: https://www.istat.it/ (Accessed August 9, 2022).

[45] Todaro Laura Francesco Romano. Impacts of Refugee flows to Territorial Development in Europe: Case Study – Integration of UAMs in Sicily, Italy. Luxembourg: EPSON (2019). 1–47.

[46] European Centre for Disease Prevention and Control. Guidance on infection prevention and control of coronavirus disease (COVID-19) in migrant and refugee reception and detention centres in the EU/EEA and the United Kingdom. Solna: European Centre for Disease Prevention and Control (2020).

[47] Prasad S Woldt J Borra H Altay N. Migrant supply chain networks: an empirically based typology. Ann Oper Res. (2020) 319:1331–58. doi: 10.1007/s10479-020-03523-w

[48] McMahon A. The Role of the State in Migration Control: The Legitimacy Gap and Moves Towards a Regional Model. Leiden: Brill | Nijhoff (2016).

[49] Esposito M Salerno M Scoto E di Nunno N Sessa F. The impact of the COVID-19 pandemic on the practice of forensic medicine: an overview. Healthcare. (2022) 10:319. doi: 10.3390/healthcare10020319, PMID: 35206933 PMC8871677

[50] Freedman J. Immigration, Refugees and responses. JCMS. (2021) 59:92–102. doi: 10.1111/jcms.13258

[51] Tosti ME Marceca M Eugeni E D’Angelo F Geraci S Declich S. Health assessment for migrants and asylum seekers upon arrival and while hosted in reception centres: Italian guidelines. Health Policy. (2021) 125:393–405. doi: 10.1016/j.healthpol.2020.12.010, PMID: 33461797

[52] Orcutt M Patel P Burns R Hiam L Aldridge R Devakumar D. Global call to action for inclusion of migrants and refugees in the COVID-19 response. Lancet. (2020) 395:1482–3. doi: 10.1016/S0140-6736(20)30971-5, PMID: 32334651 PMC7180034

[53] Fernández O Kang S, Laily Noor Ikhsanto jurusan teknik mesin, Aceh kue tradisional khas. Indicazioni operative ad Interim per la gestione di strutture con persone ad elevata fragilità e marginalità Socio-Sanitaria nel Quadro dell’epidemia di COVID-19. Presidenza del Consiglio dei Ministri Italy, Rome (2020) 2017:1–9.

[54] Fabris S d’Ettorre G Spagnolello O Russo A Lopalco M D’Agostino F. SARS-CoV-2 among migrants recently arrived in Europe from low- and middle-income countries: containment strategies and special features of Management in Reception Centers. Front Public Health. (2021) 9:735601. doi: 10.3389/fpubh.2021.735601, PMID: 34917571 PMC8669389

[55] Guenezan J Garcia M Strasters D Jousselin C Lévêque N Frasca D. Povidone iodine mouthwash, gargle, and nasal spray to reduce nasopharyngeal viral load in patients with COVID-19: a randomized clinical trial. JAMA Otolaryngol Head Neck Surg. (2021) 147:400–1. doi: 10.1001/jamaoto.2020.5490, PMID: 33538761 PMC7863011

[56] Biancolella M Colona VL Mehrian-Shai R Watt JL Luzzatto L Novelli G. COVID-19 2022 update: transition of the pandemic to the endemic phase. Hum Genomics. (2022) 16:19. doi: 10.1186/s40246-022-00392-1, PMID: 35650595 PMC9156835

[57] Are EB Song Y Stockdale JE Tupper P Colijn C. COVID-19 endgame: from pandemic to endemic? Vaccination, reopening and evolution in low- and high-vaccinated populations. J Theor Biol. (2023) 559:111368. doi: 10.1016/j.jtbi.2022.111368, PMID: 36436733 PMC9686052

